# An investigation of early enteral nutrition provision in major burn patients in Australia and New Zealand

**DOI:** 10.1111/1747-0080.12746

**Published:** 2022-06-28

**Authors:** Rochelle Kurmis, Caroline Nicholls, Yvonne Singer, Dale W. Edgar, Fiona M. Wood, Belinda J. Gabbe, Lincoln M. Tracy

**Affiliations:** ^1^ Adult Burns Service Royal Adelaide Hospital Adelaide South Australia Australia; ^2^ Department of Nutrition and Dietetics Concord Repatriation General Hospital Concord New South Wales Australia; ^3^ Victorian Adult Burns Service Alfred Hospital Melbourne Victoria Australia; ^4^ State Adult Burn Unit Fiona Stanley Hospital Murdoch Western Australia Australia; ^5^ Burn Injury Research Node The University of Notre Dame Fremantle Western Australia Australia; ^6^ Burn Injury Research Unit University of Western Australia Perth Western Australia Australia; ^7^ School of Public Health and Preventive Medicine Monash University Melbourne Victoria Australia; ^8^ Health Data Research UK, Swansea University Medical School Swansea University Swansea UK

**Keywords:** burns, enteral nutrition, parenteral nutrition

## Abstract

**Aims:**

Early enteral nutrition (provided within 24 h of admission) is the optimal form of nutritional support for major burn injuries. The aim of this study was to (i) audit early enteral nutrition practices, (ii) identify characteristics of patients who received early enteral nutrition, and (iii) investigate whether early enteral nutrition was associated with in‐hospital outcomes.

**Methods:**

An analysis of prospectively collected data from the Burns Registry of Australia and New Zealand was conducted. Specifically, this study focused on major burns patients (defined as burns affecting more than 20% and 15% total body surface area for adult paediatric patients, respectively) admitted to a specialist burn service between 1 July 2016 and 30 June 2019.

**Results:**

Data from 474 major burns patients (88 paediatric patients) revealed 69% received early enteral nutrition. Paediatric patients who received early enteral nutrition were younger than their counterparts who did not receive the same support (*p* = 0.04). Adult patients who received early enteral nutrition sustained larger burns (*p* < 0.001). Early enteral nutrition was not associated with in‐hospital mortality following major burn injury in adult patients in either unadjusted (*p* = 0.77) or confounder‐adjusted (*p* = 0.69) analyses*.*

**Conclusions:**

Approximately two‐thirds of patients with major burn injuries received early enteral nutrition. Early enteral nutrition was not associated with in‐hospital mortality following major burn injury. Further research should focus on modifiable reasons why major burns patients do not receive enteral nutrition within 24 h of admission.

## 1 INTRODUCTION

Nutritional support following major burns (defined as burns affecting more than 20% total body surface area for adult and 15% total body surface area for paediatric patients) is a recognised intervention to support the hypermetabolic response initiated as a result of the inflammatory and endocrine stress responses post‐injury.^1^ The initiation of early enteral nutrition (commencing within 24 h of admission) is considered the optimal form of nutritional support for major burn injuries^1^, ^2^, ^3^ requiring resuscitation. Early enteral nutrition significantly decreases mortality, length of stay, and in‐hospital complications such as gastrointestinal haemorrhage, sepsis, and pneumonia.^4^ The provision of nutritional support following burn injuries is affected by age, gender, baseline anthropometric measurements and nutritional status, and the size and severity of injury.^1^, ^5^ Whilst the importance of enteral nutrition following burn injury is internationally acknowledged as a component of optimal treatment,^1^, ^3^, ^5^ variations in the provision of nutrition support following burn injury in practice have been reported.^6^, ^7^, ^8^ The effects of this variation on patient outcomes are unknown.

Registry data is one way to investigate variations in nutrition support practices and the impact of variations on patient outcomes. Castanon et al. demonstrated improved outcomes in geriatric burn injuries associated with early enteral nutrition.^9^ There is a lack of high quality, multicentre nutrition outcome evidence for adults who have sustained burn injuries,^4^ and the use of burn registry data may assist in bridging this gap. The aim of this study was to: (i) audit early enteral nutrition practices in paediatric and adult burn patients; (ii) identify characteristics of patients who received enteral nutrition, and (iii) investigate whether early enteral nutrition was associated with in‐hospital outcomes (i.e., mortality, discharge disposition, and length of stay) in major burns patients in Australia and New Zealand, using data from the Burns Registry of Australia and New Zealand (BRANZ). It was hypothesised that early enteral nutrition would improve in‐hospital outcomes.

## 2 METHODS

Since July 2016, the BRANZ has collected the following data regarding enteral/parenteral feeding^10^:

(i) ‘Did the patient receive enteral or parenteral nutrition during their admission?’; and
(ii) ‘For an adult with a burn with equal to or greater than 20% total body surface area or a child with a burn equal to or greater than 15% total body surface area was enteral or parenteral feeding commenced within 24 h of admission to the burns service?’

Although the aforementioned data item covers both enteral and parenteral nutrition, hereon enteral nutrition will be used for simplicity's sake in the remainder of the manuscript. Ethical approval for the registry and related research activities were approved by the Monash University Human Research Ethics Committee (reference CF08/2431‐2008001248).

Acute admissions data for patients with major burns between 1 July 2 016 and 30 June 2 019 were extracted from the BRANZ. Patients were excluded from the study if: (a) they were transferred between two BRANZ hospitals, (b) they received end‐of‐life care on arrival to the BRANZ hospital as their burn was assessed as non‐survivable, (c) the cause of their burn was sunburn and they did not require a wound management procedure in theatre, or (d) their age could not be calculated. Intersex patients or patients of indeterminate gender were excluded due to very low volumes in the sample (<0.1%). Patients with missing or invalid burn size data were also excluded.

Age at injury was calculated using date of birth and date of injury data. Patients were stratified as either paediatric (<15 years) or adult (≥16 years) cases. This represents the age of transition from paediatric to adult healthcare providers in Australia. Patients were stratified as either receiving early enteral nutrition or not receiving early enteral nutrition per their responses to the aforementioned enteral nutrition data items. The latter group included patients who did not receive enteral nutrition at any point during their admission. The primary causes of burn groups were: flame, contact, scald, and other (covering chemical, electrical, friction, radiant heat, and other non‐flame causes).^11^ Burn depth variables were recoded to determine whether the patient sustained a full thickness burn.

In‐hospital mortality was the primary outcome of interest. Time to death, discharge disposition, and hospital length of stay were evaluated as secondary outcomes.

Categorical variables were summarised by frequencies and percentages, while continuous variables were summarised with means and standard deviations or medians and interquartile ranges. Missing data were excluded from calculations. Differences in demographic characteristics, injury event details, in‐hospital management, and in‐hospital outcomes between patients who did and did not receive enteral nutrition were assessed using chi‐square tests for categorical variables and independent samples *t*‐tests or Mann–Whitney *U* tests for continuous variables. A mixed effects logistic regression model (accounting for the random effects of the contributing burn service) was performed to determine if there was an association between receiving early enteral nutrition and in‐hospital mortality. An unadjusted model was run initially, followed by an adjusted model that accounted for confounders (variables that were associated with both early enteral nutrition and in‐hospital mortality; *p* < 0.2). Patients who died within 24 h of admission were excluded from the mixed effects logistic regression model. Unadjusted and risk‐adjusted odds ratios and 95% confidence intervals were reported. The model performance (i.e., discrimination) was assessed using the area under the receiver operating characteristic curve. According to Hosmer and Lemeshow, an area under the curve of less than 0.5 shows no discrimination, an area under the curve between 0.7 and 0.8 represents acceptable discrimination, an area under the curve between 0.8 and 0.9 shows excellent discrimination, and an area under the curve equal to or greater than 0.9 represents outstanding discrimination.^12^ Propensity matched analysis (i.e., propensity score matching) was also performed as an additional measure of sensitivity to determine the association between early enteral nutrition and in‐hospital mortality using the ‘teffects psmatch’ command. All statistical analyses were performed using Stata Version 14 (StataCorp). A *p*‐value <0.05 was considered significant.

## 3 RESULTS

**FIGURE 1:**
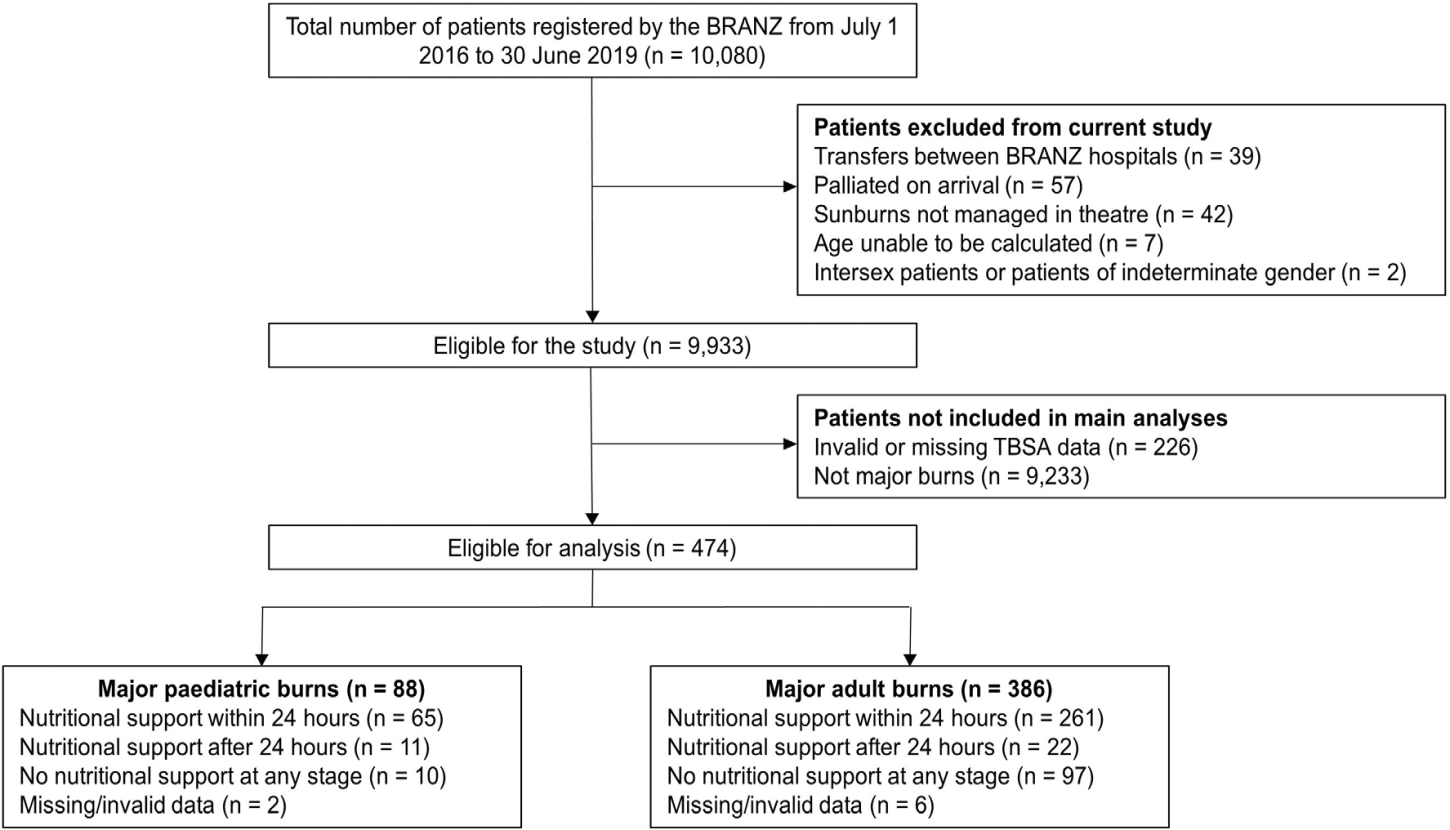
Participant flow diagram. BRANZ, Burns Registry of Australia and New Zealand; TBSA, total body surface area

There were 10 080 patients registered by the BRANZ between 1 July 2016 and 30 June 2019 (Figure 1). Four hundred and seventy‐four patients (4.7%) had major burns. There were 386 adult major burns patients (5.6% of adult patients) and 88 paediatric major burns patients (3.2% of paediatric patients; Table S1).

Of the 88 paediatric major burns included in the study, all but two had valid data on whether they received early enteral nutrition. Sixty‐five patients (73.9%) received early enteral nutrition, 11 patients (12.5%) received enteral nutrition after 24 h from admission, while 10 patients (11.4%) did not receive enteral nutrition at any point during their admission. Paediatric patients who received early enteral nutrition were younger than patients who did not receive early enteral nutrition (median age 3 years vs. 5 years, *p* = 0.04; Table 1). A greater proportion of patients who did not receive early enteral nutrition were admitted on the weekend compared to patients who received early enteral nutrition (67% vs. 38%, *p* = 0.02). There were no differences between paediatric patients who received and did not receive early enteral nutrition with respect to gender (*p* = 0.82), time to admission (*p* = 0.36), injury cause (*p* = 0.88), median burn size (*p* = 0.05), maximal recorded burn depth (*p* = 0.55), and injury intent (*p* = 0.09). The in‐hospital management of paediatric major burns patients is summarised in Table S2 (online supplementary material). Table S3 (online supplementary material) displays the in‐hospital outcomes for paediatric major burns patients by early enteral nutrition status.

**TABLE 1:** Demographic and injury event characteristics of paediatric major burns patients by early enteral nutrition status

	Early EN (*N* = 65)	No Early EN (*N* = 21)	*p*‐value
Age, median (IQR) years	3.0 (1.0, 7.0)	5.0 (2.0, 13.0)	0.04
Male	39 (60%)	12 (57%)	0.82
Admitted on weekend	25 (38%)	14 (67%)	0.02
Time from injury to admission, median (IQR) hours^a^	4.8 (1.5, 11.0)	3.4 (1.2, 7.3)	0.36
Scald	39 (60%)	13 (62%)	0.88
TBSA, median (IQR) %	25.0 (20.0, 30.0)	20.0 (18.0, 25.0)	0.05
Full thickness burn^b^	20 (33%)	4 (25%)	0.55
Unintentional injury^c^	57 (88%)	21 (100%)	0.09

*Note*: Data presented as frequency (percentage), unless otherwise specified. Excludes two patients where nutritional support data was missing or invalid. Data missing for ^a^two patients, ^b^nine patients and ^c^one patient.

Abbreviations: EN, enteral nutrition; IQR, interquartile range; TBSA, total body surface area.

Of the 386 adult major burn patients included, all but six had valid data on whether they received early enteral nutrition. Two hundred and sixty‐one patients (67.6%) received early enteral nutrition, 22 patients (5.7%) received enteral nutrition after 24 h from admission, while 97 patients (25.1%) did not receive enteral nutrition at any point during their admission. Patients who received early enteral nutrition had more severe injuries than their counterparts who did not receive the same support, as indicated by a greater median burn size (35% vs. 25%, *p* < 0.001; Table 2), a greater proportion of patients with full thickness burns (69.2% vs. 47.3%, *p* < 0.001), and a greater proportion of patients with an inhalation injury (42.4% vs. 11.3%, *p* < 0.001). A lower proportion of patients with unintentional injuries received early enteral nutrition (74.1% vs. 88.8%, *p* = 0.001). There were no differences between adult patients who received and did not receive early enteral nutrition with respect to age (*p* = 0.99), gender (*p* = 0.76), whether the patient was admitted on the weekend (*p* = 0.31), time to admission (*p* = 0.14), and injury cause (*p* = 0.74). The in‐hospital management of adult major burns patients is displayed in Table S4 (online supplementary material).

**TABLE 2:** Demographic and injury event characteristics of adult major burns patients by early enteral nutrition status

	Early EN (*N* = 261)	No Early EN (*N* = 119)	*p*‐value
Age, median (IQR) years	39.0 (28.0, 52.0)	37.0 (26.0, 55.0)	0.99
Male	207 (79.3%)	96 (80.7%)	0.76
Admitted on weekend	104 (39.8%)	54 (45.4%)	0.31
Time from injury to admission, median (IQR) hrs	4.6 (2.0, 11.0)	5.3 (2.3, 14.9)	0.14
Flame burn^a^	224 (86.2%)	101 (84.9%)	0.74
TBSA, median (IQR) %	35.0 (26.0, 50.0)	25.0 (22.5, 30.0)	<0.001
Full thickness burn^b^	171 (69.2%)	53 (47.3%)	<0.001
Inhalation injury^c^	108 (42.4%)	13 (11.3%)	<0.001
Unintentional injury^d^	192 (74.1%)	103 (88.8%)	0.001

*Note*: Data presented as frequency (percentage), unless otherwise specified. Excludes six patients where nutritional support data was missing or invalid. Data missing for ^a^1 patient, ^b^21 patients, ^c^10 patients and ^d^5 patients.

Abbreviations: EN, enteral nutrition; IQR, interquartile range; TBSA, total body surface area.

Table 3 displays the in‐hospital outcomes for adult major burns patients by early enteral nutrition status. There was no association between receiving early enteral nutrition and in‐hospital mortality (8.4% vs. 7.6%, *p* = 0.77). Of the patients who died, those who received early enteral nutrition had a greater median time to death (6.8 days vs. 0.5 days, *p* < 0.001). A greater proportion of patients who received early enteral nutrition were discharged to another hospital or healthcare facility (39.7% vs. 14.5%), rather than being discharged to their home or usual place of residence (*p* < 0.001). When considering all patients, those who received early enteral nutrition had a longer median length of stay (39.5 days vs. 17.9 days, *p* < 0.001). A similar result was demonstrated in patients who survived to discharge (38.0 days vs. 16.8 days, *p* < 0.001).

**TABLE 3:** In‐hospital outcomes for adult major burns patients by early EN status

	Early EN (*N* = 261)	No early EN (*N* = 119)	*p*‐value
Died	22 (8.4%)	9 (7.6%)	0.77
Time to death, median (IQR) days	6.8 (3.0, 21.2)	0.5 (0.3, 0.9)	<0.001
Died within 24 h of admission?			<0.001
Survived	239 (91.6%)	110 (92.4%)	
Died after 24 h	22 (8.4%)	2 (1.7%)	
Died within 24 h	0 (0.0%)	7 (5.9%)	
Discharge location			<0.001
Home	112 (46.9%)	81 (73.6%)	
Other hospital/healthcare setting	95 (39.7%)	16 (14.5%)	
Other location	32 (13.4%)	13 (11.8%)	
Hospital LOS – surviving patients, median (IQR) days^a^	39.5 (22.5, 71.7)	17.9 (13.0, 26.2)	<0.001
Hospital LOS – all patients, median (IQR) days^a^	38.0 (20.5, 69.8)	16.8 (11.3, 25.4)	<0.001

*Note*: Data presented as frequency (percentage), unless otherwise specified. Excludes two patients where nutritional support data was missing or invalid. Data missing for ^a^one patient.

Abbreviations: EN, enteral nutrition; IQR, interquartile range; LOS, length of stay.

Table 4 displays the unadjusted and adjusted mixed‐effects logistic regression models. There was no association between receiving early enteral nutrition and in‐hospital mortality after adjusting for relevant confounding factors (*p* = 0.69). The model demonstrated excellent discrimination (area under the curve 0.84, 95% CI 0.75–0.92). A second regression model based on the ‘rule of 10 events per variable’ containing early enteral nutrition, the percentage of total body surface area burned, and whether the patient sustained an inhalation injury (the first variable being our exposure of interest, the latter two variables were deemed to best account for known sources of confounding) led us to the same conclusions as our original model (Table S5, online supplementary material). The propensity score matching model (coefficient 0.0007; 95% CI −0.11 to 0.11; *p* = 0.99) also did not show an association between early enteral nutrition and in‐hospital mortality.

**TABLE 4:** Factors associated with in‐hospital mortality in adult major burns patients

	Unadjusted		Adjusted	
	OR (95% CI)	*p*‐value	OR (95% CI)	*p*‐value
Early EN		0.03		0.69
No (reference)	1.00		1.00	
Yes	5.06 (1.17–21.91)		1.39 (0.30–6.92)	
Time from injury to admission	0.56 (0.20–1.57)	0.27	–	–
TBSA	1.06 (1.03–1.08)	<0.001	1.04 (1.02–1.07)	<0.001
Full thickness burn		0.008		0.05
No (reference)	1.00		1.00	
Yes	15.12 (2.01–113.51)		8.08 (1.02–64.37)	
Inhalation injury		0.004		0.77
No (reference)	1.00		1.00	
Yes	3.63 (1.52–8.65)		1.40 (0.53–3.73)	
Unintentional injury		0.008		0.98
No (reference)	1.00		1.00	
Yes	0.31 (0.13–0.73)		0.98 (0.36–2.65)	
Theatre within 24 h of admission?		0.78		–
No theatre (reference)	1.00		–	
Not admitted within 24 h	1.45 (0.17–12.45)		–	
Admitted within 24 h	1.05 (0.13–8.48)		–	

Abbreviations: CI, confidence interval; EN, enteral nutrition; OR, odds ratio; TBSA, total body surface area.

## 4 DISCUSSION

Two thirds of all major burn patients were recorded to have received early enteral nutrition following admission to a hospital with a specialist burns service. Paediatric patients receiving early enteral nutrition were younger than patients not receiving early enteral nutrition. This may relate to adolescents being assessed as able to meet their nutrition requirements orally. Adults receiving early enteral nutrition had sustained more severe injuries than their counterparts who did not receive early enteral nutrition. There was no association between early enteral nutrition and in‐hospital mortality. However, time to death data indicated all patients who received enteral nutrition survived at least 24 h after admission. Patients who received early enteral nutrition but died survived for longer compared to patients who died without receiving early enteral nutrition. This may be reflective of their less severe injuries at admission, compared to the early mortality group, but with the potential for underlying comorbidities or concomitant trauma‐related injuries to complicate care. Patients who received early enteral nutrition remained in hospital for nearly twice as long as patients who did not receive early enteral nutrition. This is likely reflective of the groups injury severity, warranting enteral nutrition to supplement care if patients were not treated with palliative intent upon admission.

It must be acknowledged that length of stay may be considered a poor choice of outcome measure for nutritional interventions as it is influenced by multiple other factors in burn injury care. However, it was included due to the precedence for its use in other studies and the lack of more appropriate functional outcome measures within the BRANZ. The effectiveness of early enteral nutrition versus standard enteral nutrition following burn injury in adults remains unclear.^13^ Early initiation of enteral nutrition following burn injury, however, has multiple significant benefits including the preservation of gastrin secretion and motility of the gastrointestinal tract, reduced intestinal permeability, and maintenance of mucosal barrier function,^14^ as well as fewer infections complications.^4^, ^15^ This is particularly important following burn injuries, as burns patients have higher risks of enteral feed intolerance when compared to other critical care populations.^16^ Enteral feed intolerance has been associated with worse clinical outcomes, including increased hospital length of stay.^16^ Enteral nutrition support has also been linked to decreased hospital‐acquired infections, resulting in a decreased length of stay **of** 4.7 days and significant health care cost savings, within US Medicare.^17^, ^18^

In the meta‐analysis conducted by Pu et al., early enteral nutrition was associated with a decreased rate of mortality and a reduced length of stay.^4^ The findings of the current study were inconsistent with those of the earlier meta‐analysis, with no association observed between early enteral nutrition and mortality, and patients who received early enteral nutrition having a longer length of stay. However, the studies included in the Pu et al. meta‐analysis did not specify how in‐hospital outcomes were classified. Consequently, it is difficult to comment on if and how these differences contributed to the inconsistent findings of the current study.

The present study's findings of a rate of 30% of major burns patients not receiving early enteral nutrition is higher than the 20% reported by Mosier et al. in their multi‐centre trial findings.^15^ However, Mosier et al. only included mechanically ventilated adult patients. This may partially explain why the number of patients not receiving early enteral nutrition increased from one in five to one in three in the present study, as mechanically ventilated patients are more likely to receive early enteral nutrition compared to patients capable of consuming food orally. Mosier et al. were unable to identify logistical, patient, injury, or resuscitation barriers to early enteral nutrition initiation. They proposed provider factors accounted for the variation in early enteral nutrition practices, but did not define or elaborate on what such factors were. The 30% rate is also higher than the 26% rate reported from a 2012 audit of Australian and New Zealand burns services.^7^ One reason for not complying with enteral nutrition timing targets was patients being admitted on a weekend (and not being referred to the dietitian until the next weekday) or enteral nutrition not being indicated despite the large burn size (12% of patients). Another reason was patients being able to meet nutritional requirements with oral nutrition support or refusal of enteral feeding tube (26%).^7^ These factors, combined with practice variations between services, likely underlies the variance observed in the current study.

The current study's finding that adult patients who received early enteral nutrition had more severe injuries was somewhat inconsistent with Mosier et al., where patients receiving early enteral nutrition only had a smaller mean burn size.^15^ However, the difference in inclusion criteria between studies must be noted here as Mosier et al. only included mechanically ventilated patients aged 18 years and older who were admitted within 96 h of injury; none of these criteria applied to the current study. In this study, a greater proportion of adult patients who received early enteral nutrition support had full thickness burns, documented suspicion of an inhalation injury, and a greater median burn size.

Utilisation of BRANZ data allowed for comparison of all patients with a major burn injury admitted to a specialist burns service within Australia and New Zealand. This allowed for a larger cohort of patients in this analysis than what would have been feasible in a prospective design. Data analysis was retrospective, which limited the ability to make causal associations. The BRANZ does not collect any information on the quality or quantity of enteral nutrition provided to patients, nor does it collect data on enteral feed intolerance. Consequently, it was not possible to investigate how enteral nutrition could be optimised (or what the available data tells us) beyond the timing of when enteral nutrition was initiated. The BRANZ also does not collect data in a way that allows for differentiation between patients who receive enteral or parenteral feeding. It is recommended that future revisions of the BRANZ include a data item to identify these separate groups of patients. Additionally, the small number of paediatric admissions limited whether associations between early enteral nutrition and outcomes could be determined. Finally, as the BRANZ does not collect data on why enteral nutrition timing targets were not met, comparison of justification with the prior audit is precluded. The BRANZ should consider creating additional data fields to track feed intolerance, timings and amounts.

Whilst early enteral nutrition initiation rates following severe burn injury were comparable to previous reports, they remain sub‐optimal. The smaller proportion of paediatric patients admitted on the weekend who received early enteral nutrition may reflect a lack of dietetics service coverage in burns services. As no such difference was observed in the adult cohort, it may also reflect differences in feeding protocols and practices for weekend admissions between paediatric and adult burn services. However, adult data suggests early enteral nutrition was appropriately commenced in patients with more severe injuries. This may explain why groups receiving early enteral nutrition demonstrated a longer hospital length of stay. Furthermore, the data suggest that for patients who were deemed to have non‐survivable injuries (a contraindication to enteral nutrition), enteral nutrition was correctly not initiated as per standard protocols. The authors conclude that the current study's results are due to clinical, not statistical, bias arising from routine and consistent clinical practices for burn care within Australia and New Zealand. The Emergency Management of Severe Burns course (developed and taught by the Australian and New Zealand Burn Association to all health professionals who provide care to patients with an acute and severe burn injury) outlines that all patients with a burn affecting 20% or more of their body receive enteral nutrition *prior* to a decision to engage palliative management is made.^19^ Consequently, all life‐threatening burns in Australia and New Zealand should follow this treatment decision pathway.

Two‐thirds of patients with major burn injuries received early enteral nutrition following admission to a specialist burn service. Early enteral nutrition is appropriately commenced in patients with severe but survivable injuries and withheld from patients displaying contraindications, such as non‐survivable injuries. Early enteral nutrition was not associated with in‐hospital mortality. Future research into the reasons why major burns patients did not receive enteral nutrition would be beneficial.

## AUTHOR CONTRIBUTIONS

RK, CN, and LMT contributed to the conception and design of the research. BJG and LMT contributed to the analysis of data. RK, CN, YS, DWE, FMW, and BJG contributed to the interpretation of the data. RK, CN, and LMT drafted the manuscript. All authors critically revised and approved the final manuscript. The authors acknowledge the BRANZ Steering Committee for their support of this project and for the provision of data.

## CONFLICT OF INTEREST

The funding sources had no involvement in the study design, the collection, analysis, and interpretation of data, in the writing of the report, and in the decision to submit the article for publication. The authors declare no conflicts of interest.

## Supporting information


**Table S1** Demographic and injury characteristics of adult and paediatric major burns patients
**Table S2**: In‐hospital management of paediatric major burns patients by early EN status
**Table S3**: In‐hospital outcomes for paediatric major burns patients by early EN status
**Table S4**: In‐hospital management of adult major burns patients by early EN status
**Table S5**: Additional regression model for in‐hospital mortality (1:10 EPV rule)

## Data Availability

The data are not publicly available due to privacy or ethical restrictions. Information about requesting access to BRANZ data can be found at https://www.monash.edu/medicine/sphpm/branz/data-requests.
