# Supplementary material for: An investigation of early enteral nutrition provision in major burn patients in Australia and New Zealand
Source: Nutr Diet. 2022 Jun 28;79(5):582–9. doi: 10.1111/1747-0080.12746 (PMC9796319; doi:10.1111/1747-0080.12746)
Supplement: Supplementary file 1 — Table S1 Demographic and injury characteristics of adult and paediatric major burns patients Table S2: In‐hospital management of paediatric major burns patients by early EN status Table S3: In‐hospital outcomes for paediatric major burns patients by early EN status Table S4: In‐hospital management of adult major burns patients by early EN status Table S5: Additional regression model for in‐hospital mortality (1:10 EPV rule) [file NDI-79-582-s001.docx]

**SUPPLEMENTARY MATERIALS**

| **Table S1: Demographic and injury characteristics of adult and paediatric major burns patients** | | |
| --- | --- | --- |
|  | **Paediatric** | **Adult** |
| N | 88 | 386 |
| Age, median (IQR) | 4.0 (1.0, 9.0) | 39.0 (27.0, 53.0) |
| Gender |  |  |
| Male | 53 (60%) | 308 (79.8%) |
| Female | 35 (40%) | 78 (20.2%) |
| Time from injury to admission (hours), median (IQR)^a^ | 4.7 (1.3, 10.2) | 4.7 (2.0, 11.7) |
| Admitted within 24 hours of injury?^b^ | 77 (90%) | 343 (88.9%) |
| Primary Cause^c^ |  |  |
| Flame | 31 (35%) | 330 (85.7%) |
| Scald | 54 (61%) | 28 (7.3%) |
| Contact | < 5 (2%) | 7 (1.8%) |
| Other cause | < 5 (< 5%) | 20 (5.2%) |
| %TBSA, median (IQR) | 22.6 (19.0, 29.0) | 30.0 (24.5, 44.0) |
| Superficial burn only^d^ | < 5 (< 5%) | 13 (3.9%) |
| Full thickness burn^e^ | 25 (32%) | 228 (62.5%) |
| Inhalation injury^f^ | < 5 (< 5%) | 123 (32.9%) |
| Injury Intent^g^ |  |  |
| Unintentional | 80 (91%) | 301 (79.0%) |
| Intentional, self-harm | < 5 (< 5%) | 52 (13.6%) |
| Intentional, other | < 5 (< 5%) | 13 (3.4%) |
| Other or undetermined intent | < 5 (< 5%) | 15 (3.9%) |
| Data presented as frequency (percentage), unless otherwise specified.  Data missing for ^a^2 patients, ^b^2 patients, ^c^1 patient, ^d^64 patients, ^e^31 patients, ^f^13 patients, and ^g^5 patients.  IQR = interquartile range; TBSA = total body surface area. | | |

| **Table S2: In-hospital management of paediatric major burns patients by early EN status** | | | |
| --- | --- | --- | --- |
|  | **Early EN**  **(N = 65)** | **No Early EN**  **(N = 21)** | **p-value** |
| Procedure in theatre | 65 (100%) | 19 (90%) | 0.012 |
| Time to first theatre (hours), median (IQR) hours^a^ | 18.2 (5.8, 86.2) | 14.7 (1.9, 78.7) | 0.69 |
| Theatre within 24 hours of admission?^a^ | 36 (61%) | 12 (63%) | 0.87 |
| Theatre within 48 hours of admission?^a^ | 38 (64%) | 13 (68%) | 0.75 |
| ICU admission | 35 (54%) | 4 (19%) | 0.005 |
| Time to first ICU admission, median (IQR) hours^b^ | 2.6 (0.1, 8.3) | 5.6 (2.5, 42.6) | 0.30 |
| Nutritional status screened within 24 hours?^c^ | 60 (97%) | 15 (79%) | 0.009 |
| Data presented as frequency (percentage), unless otherwise specified.  Excludes two patients where nutritional support data was missing or invalid. Data missing for ^a^6 patients, ^b^1 patient, and ^c^5 patients.  EN = enteral nutrition; ICU = intensive care unit; IQR = interquartile range. | | | |

| **Table S3: In-hospital outcomes for paediatric major burns patients by early EN status** | | | |  |
| --- | --- | --- | --- | --- |
|  | **Early EN**  **(N = 65)** | **No Early EN**  **(N = 21)** | **p-value** | |
| Discharged to home or usual residence | 50 (77%) | 20 (95%) | 0.061 | |
| Hospital LOS, median (IQR) days^a^ | 26.9 (17.4, 47.8) | 17.7 (9.8, 37.6) | 0.029 | |
| Data presented as frequency (percentage), unless otherwise specified.  Excludes two patients where nutritional support data was missing or invalid. Data missing for ^a^1 patient.  EN = enteral nutrition; IQR = interquartile range; LOS = length of stay; MRO = multidrug resistant organism. | | | |  |

| **Table S4: In-hospital management of adult major burns patients by early EN status** | | | |
| --- | --- | --- | --- |
|  | **Early EN**  **(N = 261)** | **No Early EN**  **(N = 119)** | **p-value** |
| Procedure in theatre | 253 (96.9%) | 101 (84.9%) | <0.001 |
| Time to first theatre, median (IQR) hours^a^ | 12.8 (4.3, 37.5) | 17.0 (5.2, 36.8) | 0.17 |
| Theatre within 24 hours of admission?^a^ |  |  | <0.001 |
| No theatre admission | 8 (3.2%) | 18 (15.5%) |  |
| Not admitted within 24h | 78 (30.8%) | 35 (30.2%) |  |
| Admitted within 24h | 167 (66.0%) | 63 (54.3%) |  |
| Theatre within 24-48 hours of admission?^a^ |  |  | 0.18 |
| No theatre admission | 216 (88.2%) | 81 (82.7%) |  |
| Admitted within 24-48 hours of admission | 29 (11.8%) | 17 (17.3%) |  |
| Theatre within 48 hours of admission?^a^ |  |  | <0.001 |
| No theatre admission | 8 (3.2%) | 18 (15.5%) |  |
| Not within 48h | 49 (19.4%) | 18 (15.5%) |  |
| Admitted within 48h | 196 (77.5%) | 80 (69.0%) |  |
| ICU admission^b^ | 220 (84.3%) | 40 (33.9%) | <0.001 |
| Time to first ICU admission, median (IQR) hours^c^ | 0.0 (0.0, 4.6) | 1.7 (0.0, 5.5) | 0.20 |
| ICU within 24 hours of admission?^c^ |  |  | <0.001 |
| No ICU admission | 41 (15.9%) | 78 (66.1%) |  |
| Not admitted within 24h | 6 (2.3%) | 7 (5.9%) |  |
| Admitted within 24h | 211 (81.8%) | 33 (28.0%) |  |
| ICU within 48 hours of admission?^c^ |  |  | <0.001 |
| No ICU admission | 41 (15.9%) | 78 (66.1%) |  |
| Not within 48h | 4 (1.6%) | 7 (5.9%) |  |
| Admitted within 48h | 213 (82.6%) | 33 (28.0%) |  |
| Mechanical ventilation within 24 hours of admission?^d^ | 121 (97.6%) | 15 (93.8%) | 0.39 |
| Mechanical ventilation in ICU^e^ | 195 (89.9%) | 24 (61.5%) | <0.001 |
| Malnutrition risk status screened within 24 hours?^f^ | 235 (94.4%) | 93 (81.6%) | <0.001 |
| Data presented as frequency (percentage), unless otherwise specified.  Excludes six patients where nutritional support data was missing or invalid. Data missing for ^a^11 patients, ^b^1 patient, ^c^3 patients, ^d^1 patient, ^e^4 patients, and ^f^17 patients.  EN = enteral nutrition; ICU = intensive care unit; IQR = interquartile range. | | | |

An additional model based on the “rule of ten events per variable” is included to provide confidence that the estimates in the original model have not been biased by the inclusion of more variables than the 1:10 EPV rule. The new model contains three variables: whether the patient received enteral nutrition within 24 hours of admission, the total body surface area (TBSA) burned, and whether clinical documentation indicated the patient sustained an inhalation injury in addition to their cutaneous burn. The first variable was selected as this is our exposure of interest. The latter two variables were selected as they were deemed to best account for known sources of confounding. Specifically, inhalation injury often requires intubation and mechanical ventilation regardless of the size of the accompanying cutaneous burn. Infections are a leading cause of mortality in burn injury, and pneumonia is a life threatening infectious complication often associated with inhalation injury and/or endotracheal intubation. The TBSA burned is an important consideration of nutritional management for the severely burned patient, irrespective of whether inhalation injury is also present. Severe burn injuries lead to a hypermetabolic response that is typically proportional to the size of the burn and is characterised by inflammatory and endocrine stress responses (i.e., the bigger the burn, the greater the hypermetabolic response, and the greater need for nutritional management and support following administration). Inhalation injury and increasing burn size have also been identified as key predictors of in-hospital mortality following burn injury.

| **Table S5: Additional regression model for in-hospital mortality (1:10 EPV rule)** | | |
| --- | --- | --- |
|  | **OR (95% CI)** | ***p*-value** |
| Early EN |  | 0.42 |
| No (reference) | 1.00 |  |
| Yes | 1.90 (0.40, 9.12) |  |
| TBSA | 1.05 (1.02, 1.07) | <0.001 |
| Inhalation injury |  | 0.17 |
| No (reference) | 1.00 |  |
| Yes | 1.93 (0.75, 4.96) |  |
| CI = confidence interval; EN = enteral nutrition; EPV = events per variable; OR = odds ratio; TBSA = total body surface area. | | |
